# Plasma Streamwise Vortex Generators for Flow Separation Control on Trucks: A Proof-of-concept Experiment

**DOI:** 10.1007/s10494-018-9891-9

**Published:** 2018-02-12

**Authors:** Julie A. Vernet, Ramis Örlü, David Söderblom, Per Elofsson, P. Henrik Alfredsson

**Affiliations:** 1 Linné FLOW Centre, KTH Mechanics, SE-100 44 Stockholm, Sweden; 2 Scania CV AB, SE-15187 Södertälje, Sweden

**Keywords:** Vehicle aerodynamics, Separation control, Plasma actuation

## Abstract

An experimental study of the effect of Dielectric Barrier Discharge plasma actuators on the flow separation on the A-pillar of a modern truck under cross-wind conditions has been carried out. The experiments were done in a wind tunnel with a 1:6 scale model of a tractor-trailer combination. The actuators were used as vortex generators positioned on the A-pillar on the leeward side of the tractor and the drag force was measured with a wind-tunnel balance. The results show that the effect at the largest yaw angle (9 degrees) can give a drag reduction of about 20% and that it results in a net power reduction. At lower yaw angles the reduction was smaller. The present results were obtained at a lower Reynolds number and a lower speed than for real driving conditions so it is still not yet confirmed if a similar positive result can be obtained in full scale.

## Introduction

Efficient aerodynamic design of ground vehicles in general and heavy-duty trucks in particular is an important factor to reduce drag and thereby fuel consumption. In the strive towards a fossil-free transport sector such a reduction is even more important since in most cases the cost of future fuels will be higher and the power density lower.

On heavy duty trucks better designs of both the front of the tractor and the tail of the trailer have potential to reduce drag [see e.g. 1, 2]. An example is the flow around the A-pillar of the tractor where considerable development work has been done to find an optimal rounded shape. It has been demonstrated that such a design can decrease the drag coefficient considerably as compared to box-shaped vehicles [3]. However, the design is optimal for a specified flow direction, i.e. a direct head wind, but vehicles are in most cases not moving into still air or head winds, but there exists a cross wind that gives an effective yaw angle. It is also well known that the drag on a tractor-trailer combination increases substantially under yawed conditions. For such cases it may be useful to have an active flow control strategy that can adapt flow control measures to the specific circumstances.

Dielectric Barrier Discharge (DBD) plasma actuators have shown effectiveness in controlling flow separation [4, 5] e.g. on geometries such as airfoils or cylinders. In some studies they have been used to control flow separation and reduce drag by ‘injecting’ momentum in the streamwise direction close to the separation line [see e.g. 6]. However, in the aforementioned study they were found to only be capable of reducing the size of the recirculation bubble for freestream velocities close to the electric wind velocity which in that study reached speeds of a few meters per second. Furthermore, it was shown that the effect of the actuation was strongly dependent on the relative position between the separation line and the position of the actuator, which could lead to difficulties when applying the technique to flow cases of non-fixed separation position. To overcome these limitations another configuration of the plasma actuators has been suggested, namely that of an array of DBD plasma actuators creating streamwise vortices similar to classical vortex generators [see e.g. 7, 8]. This is also the approach taken here and to the best of the authors knowledge, this is the first time DBD-VGs have been tested to control flow separation on a realistic tractor geometry. For the present proof-of-concept application, an array of DBD-VGs has been mounted on a truck model and balance measurements have been performed to directly assess the drag under no-control and control conditions for various velocities and yaw angles.

The measurements were carried out during one and half day in the Lola Cars wind tunnel in the UK. Therefore the results have to be viewed as preliminary, and the time allotted did not allow a wide variation of the parameters with regard to the plasma actuators. However, the results clearly show that it was possible to achieve a substantial reduction in drag, especially at high yaw angles. For some cases this could be translated to a net power reduction even when taking the power consumption of the actuator into account. Therefore further studies of the use of DBD-VG plasma actuators should be carried out in order to prepare for future implementation of this technique to reduce drag and thereby fuel consumption on heavy-duty trucks.

## Experimental Set-up

**Fig. 1:**
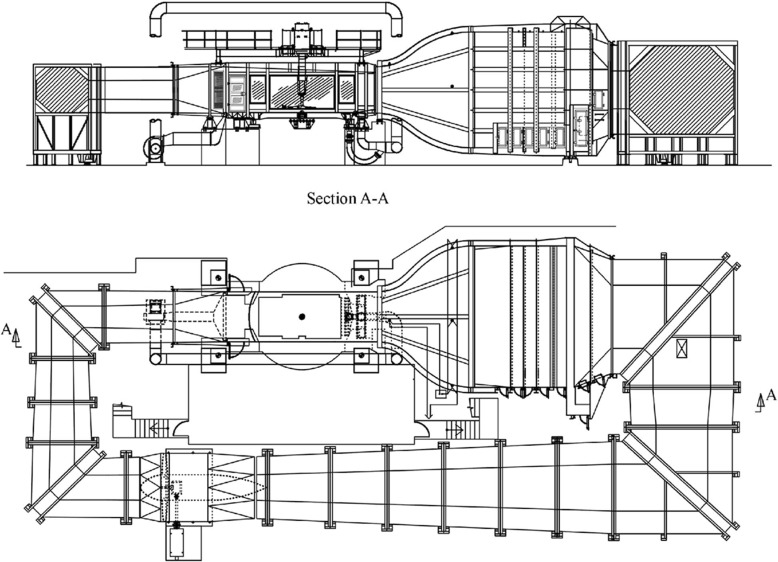
Drawing of the “Lola Cars International 50% Scale Wind Tunnel”. Reprinted from [9]. Reprinted with permission by SAE Ⓒ 2000 SAE International. Further distribution of this material is not permitted without prior permission from SAE

The measurements were conducted in the Lola Cars wind tunnel in Huntingdon, United Kingdom; see the sketch in Fig. 1 for an outline of the wind tunnel. This closed-loop wind tunnel was designed to be able to conduct aerodynamic force measurements on 1:2 scale race cars running at a maximum speed of 60 m/s with a turbulence intensity lower than 0.07% [9]. The wind tunnel has also been used for more than 10 years by Scania CV AB for aerodynamic development of heavy duty trucks. The large test section of 2.7×2.47 m^2^ (width and height, respectively) allows measurements at a low blockage ratio when using a 1:6 scale model of a generic Scania truck. The model has a projected frontal area (*A*) of 0.289 m^2^, which gives a blockage lower than 5% at zero yaw angle. Although the blockage ratio is quite small, in order to obtain correct absolute drag measurements one usually adopts a blockage correction, however, for the purpose of the present study we are mainly interested in relative changes between the case with and without control and therefore such a correction is not necessary. The total length of the tractor and trailer is 2.75 m.

The ground floor of the test section consists of a turntable, that can be rotated with an angle of up to ± 10° to also allow studies on yawed models, i.e. under cross-wind conditions. In the following the yaw angle is denoted by *α*. A 2 m wide and 4 m long rolling road system, commonly called ‘moving belt’, is installed in the central part of the turntable to reproduce the relative motion between the ground and the model. However, with actuation the belt system could not be used as the power cables for the plasma actuators had to be taped on the bottom floor of the test section, hence on the belt. This also limited the usable speed of the wind tunnel to 40 m/s, since at higher speeds the belt started to lift from the ground plate. In front of the belt there is a suction slot which sucks out the boundary layer that forms on the ground floor. The suction was in use for all cases, both those with the moving belt and the ones where the belt was stationary.

**Fig. 2:**
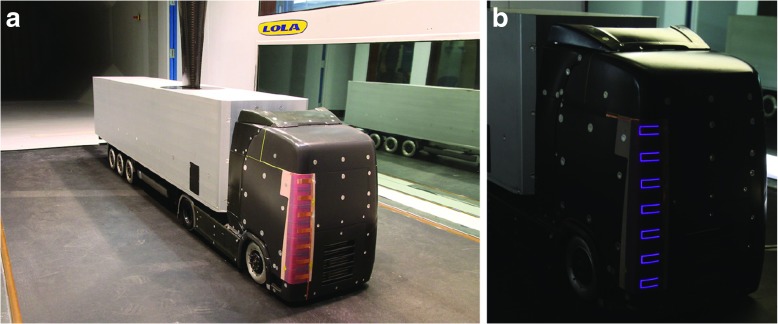
**a** Picture from the wind tunnel experiments at the Lola cars aerodynamic wind tunnel with the truck model equipped with an array of DBD-VGs without the cabling and **b** a zoom-in of the right A-pillar during actuation. The violet light is typical of these plasmas

The wind tunnel was developed for force measurements using a six-components balance that is located above the test section. The balance is connected to the model through a strut coming down into the test section; the strut attached to the top of trailer of the model is visible in Fig. 2. The balance is mounted on its own mechanical support to be isolated from the wind tunnel. The zero reading of the balance was taken before each measurement run.

The truck model used had a generic exterior design based on a Scania chassis and had interchangeable A-pillars to allow easy mounting of the DBD-VGs. The plasma array was mounted on the right A-pillar and the measurements were made at four yaw angles in the interval 0° to 9° so that the flow separation would be expected on the A-pillar on the right-hand side of the chassis, which in this case corresponds to the leeward side of the tractor, see Fig. 2b. The DBD-VG plasma actuators were made of 66 *μ* m-thick copper electrodes applied on both sides of a 396 *μ* m-thick dielectric sheet made of Kapton and Teflon layers. In short, they produce pairs of counter-rotating vortices and the actuation wavelength is 55 mm, which was chosen based on parametric studies on passive VGs [10]. Further details on the in-house built actuators and related wind-tunnel studies to decide the actuation parameters can be found in Ref. [8]. In this experiment a total of 14 vortices were introduced by the actuators, seven with clockwise and seven with counter-clockwise rotation. In Fig. 2a the placement of the actuators on the A-pillar can be seen, whereas a close-up during actuation is shown in subplot b). In this study the three different (peak-to-peak) actuation driving voltages (*V*_*d*_), 8.5, 10, and 12 kV_*p*−*p*_ were used. In all cases the driving frequency was 6.5 kHz. One should note that the right-hand side A-pillar was trimmed to be able to flush mount the actuators and only the exposed electrodes of the actuator were protruding from the surface, hence both A-pillars had the same shape.

The data were collected by the tunnel measurement system, where each measurement point was sampled and averaged for 30 seconds. Many of the measurement cases were conducted several times to ensure repeatability. In case of several measurements available the ensemble average of the cases will be presented in the figures and errors bars indicate the standard deviation.

## Results and Discussion

### Power consumption of plasma actuators

In all active flow control situation it is necessary that the penalty of the flow control itself is smaller than the positive effect it contributes to. For plasma actuators the penalty is in form of the power consumption of the actuators, *P*_*act*_ and also the parasitic power consumption by the electronics and cables powering the actuators. In the present case the power consumption of the DBD-VG plasma actuators was obtained from readings of the voltage and current from the laboratory low-voltage generator used to power the high-frequency, high-voltage generator of type *Minipuls2* (*GBS Elektronik*; see Vernet et al. [8]) and thereby the plasma actuators. In the case of drag reduction one may introduce the so called power-gain coefficient Γ defined as

1 $$ {\Gamma}=\frac{P_{saved}}{P_{act}}=\frac{U_{0} {\Delta} D}{P_{act}}\, , $$

where *P*_*saved*_ is the decrease in power consumption due to the control and is expressed as the inlet (vehicle) velocity *U*_0_ times the difference in drag Δ*D* [see 11]. If the ratio is larger than one, i.e. Γ > 1, a net gain has been obtained.

**Fig. 3:**
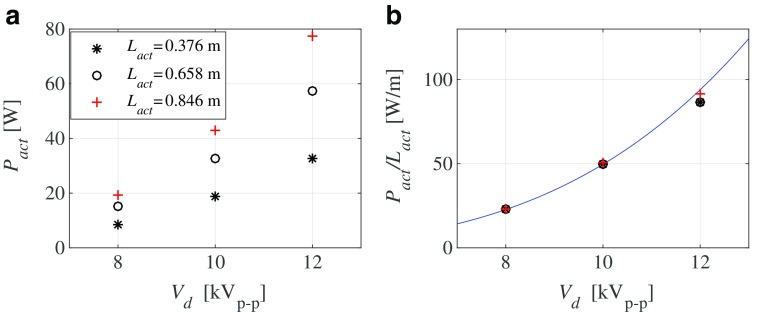
**a** Actuation power *P*_*act*_ as function of driving voltage *V*_*d*_ for different actuation length *L*_*act*_ of the plasma array. **b** Scaled actuation power for the same data as in *a)*. Solid line represents the relation $P_{act}/L_{act}= 0.0157 {V_{d}}^{3.5}$

Power measurements were done for actuators with different length of actuation (see Fig. 3a) which allowed us to determine the parasitic power losses in the high-frequency, high-voltage generator and connecting cables and obtain the power per unit length of actuation. This was done by measuring the power consumption for different actuator lengths for a given peak-to-peak voltage and from these data it was possible to obtain the parasitic power loss (which was a few watts). Figure 3b shows the actuator power consumption obtained by subtracting the parasitic loss from the total power as function of driving voltage. Earlier results [12, 13] indicate that the plasma actuators power consumption is proportional to $V_{d}^{3.5}$ (corresponding to the solid line in Fig. 3b), a result which seems to correspond well to the power consumption of the actuators used in the present study. In the following experiments 7 actuators (corresponding to a length 0.658 m) were used and for the 12 kV_*p*−*p*_ driving voltage the parasitic power losses were less than 4% of the total power for this case.

### Drag measurements with and without control

In the following we present the drag measurements using the non-dimensional drag coefficient *C*_*D*_ defined as

2 $$ C_{D}=\frac{D}{\frac{1}{2} \rho {U_{0}^{2}} A}\, , $$

where *D* is the drag force in the direction of the vehicle, *ρ* denotes the air density and *A* is the frontal area of the truck.

**Fig. 4:**
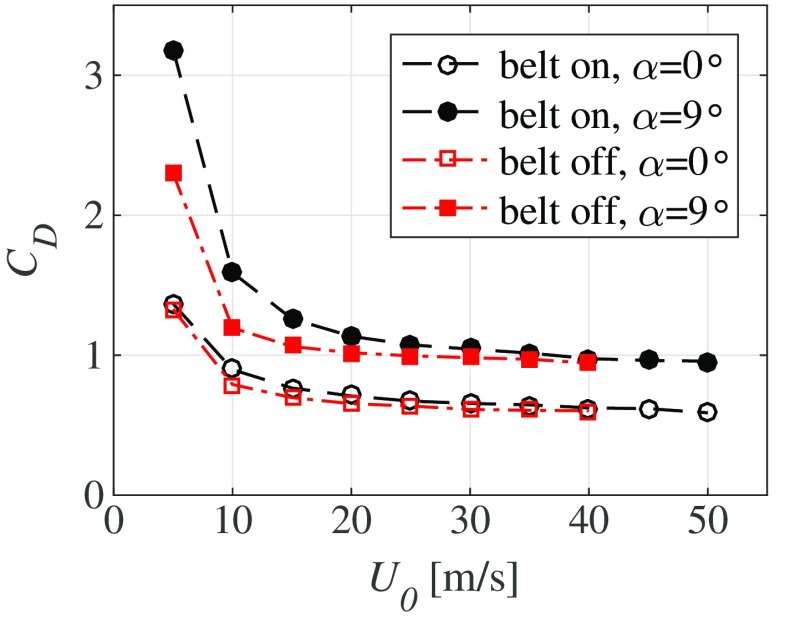
Drag coefficient *C*_*D*_ as function of freestream speed *U*_0_ for yaw angles (*α*) 0° and 9° with and without the belt running

In most wind-tunnel experiments, where bluff-body models of e.g. a vehicle or other object is studied, one strives to have a high enough Reynolds number (*Re*) so that effects of *Re* are small. This is of importance to get an accurate estimation of the full-scale drag. Model vehicle experiments are usually carried out at lower *Re* than the one in the real situation. However, as is well known, Reynolds number effects diminish as *Re* increases and usually becomes quite small if *Re* is high enough. In the case of truck aerodynamics the length scale in the Reynolds number can be based on the height of the truck or the square root of the frontal area and is about 5 million (5 ⋅ 10^6^) for a truck driving on a highway. In the Lola Cars wind tunnel typical Scania tests are carried out at speeds around 50 m/s, which corresponds to approximately twice the speed of highway driving. For 1:6 scale model the Reynolds number is hence one third of the actual full scale *Re*. As a rule of thumb a *Re* around 10^6^ should be sufficient to avoid *Re* effects, however for the present experiments we are below this value. Therefore the drag coefficient was measured with different wind-tunnel speeds and in Fig. 4 the drag coefficient is plotted versus the speed for two yaw angles (*α* = 0° and *α* = 9°) with and without the moving belt. As can be seen, above 10 m/s, *C*_*D*_ seems to be only slightly decreasing with *U*_0_, i.e. showing that no significant Reynolds number effects occur for velocities higher than 10 m/s especially without the belt running. As expected *C*_*D*_ is higher for *α* = 9° as compared to *α* = 0°.

Since the control efficiency usually decreases with increasing free-stream velocity, here measurements were carried out at the wind-tunnel speeds of 10, 15 and 20 m/s.

**Fig. 5:**
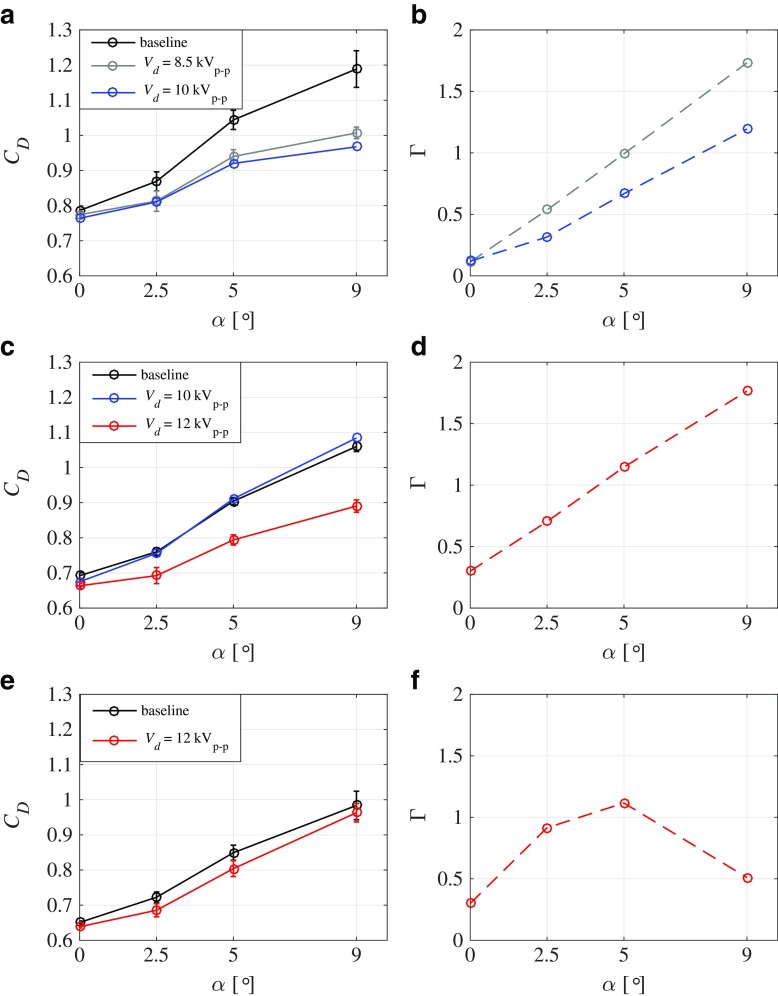
**a**, **c**, **e** Drag coefficient *C*_*D*_ as function of yaw angle *α* at a freestream speed (*U*_0_) of **a** 10, **c** 15, and **e** 20 m/s for the baseline case and controlled cases with driving voltages *V*_*d*_ of 8.5 and 10 kV_*p*−*p*_. **b**, **d**, **f** Power-gain coefficient Γ as function of angle-of-attack *α* for the controlled cases shown in **a**, **c**, **e**

The left-hand panels in Fig. 5 show the measured *C*_*D*_ values as function of the yaw angle. The three figures correspond to three different speeds, namely 10, 15 and 20 m/s. In all cases the baseline case (i.e. without control) is compared with the controlled case. As a parameter for the control the driving voltage *V*_*d*_ is varied to see how it affects the control. In the right-hand panel of these figures the power-gain coefficient (Γ) is also shown.

Figure 5 shows the results at *U*_0_ = 10 m/s and compares the baseline case with the controlled cases with *V*_*d*_ = 8.5 and 10 kV_*p*−*p*_, respectively. As can be seen the *C*_*D*_ for both control cases lies below the baseline case and increasingly so for increasing *α*. There is almost no difference between the two different *V*_*d*_ indicating that also for the low voltage in this case there is an efficient generation of the streamwise vortices, which achieve a drag reduction of up to 20% at a yaw angle of 9°. The right-hand panel shows that Γ becomes positive for both driving voltages for high *α* and that the lowest voltage actually gives the best power coefficient, i.e. there is a net power reduction. On the other hand, for small yaw angles, despite the fact that there is drag reduction, no net power decrease is obtained.

At 15 m/s (see Fig. 5c–d) only the highest driving voltage, i.e. *V*_*d*_ = 12 kV_*p*−*p*_, gives drag reduction, indicating that a *V*_*d*_ of 10 kV_*p*−*p*_ is not sufficient to create streamwise vortices that are strong enough to affect the separation. For *V*_*d*_ = 12 kV_*p*−*p*_ a net drag reduction, i.e. Γ > 1 is obtained for *α* = 5°. Also at 20 m/s (see Fig. 5e–f) drag reduction is obtained with *V*_*d*_ = 12 kV_*p*−*p*_, but the reduction is smaller than at 15 m/s and Γ is only slightly above 1 for *α* = 5°.

## Summary and Conclusions

A proof-of-concept study of the possibility to use plasma vortex generator actuators for drag reduction on heavy-duty trucks have been carried out through wind-tunnel measurements where the actuators have been placed on the A-pillar. The main goal was to see how the actuators can be used to reduce the drag when the truck travels with a yaw angle with respect to the relative wind velocity. The wind-tunnel experiments show that drag reduction is possible and at an angle of 9° a reduction of up to 20% can be achieved. In terms of the power coefficient it shows that also a net drag reduction is possible, i.e. if the penalty power consumption of the actuators are taken into account.

Although this study needs to be taken with some caution as the Reynolds number in the wind tunnel is smaller than for full scale, the results are encouraging. Since the drag scales as the frontal area of the vehicle, whereas the power consumption of an array of actuators scales linearly with the length of actuation the power-gain factor can be assumed to be higher for the full-scale case as compared to the model case. One would, however, expect the need for stronger actuation which can be obtained at higher driving voltages or with longer electrodes which would increase the power consumption of the actuation [7].

Further studies are clearly needed, both with respect to full scale experiments, but also through exploring various actuator designs and schemes. The possibility to pulse the actuation with a duty-cycle of for instance 50% actuation time would reduce the power consumption with the same amount. This possibility was investigated in Ref. [10] for jet control of separation and gave slightly lower control effect than for full time actuation, but could probably still be more efficient in terms of the net power saving. Also further development of the plasma actuators need to be done in order to secure a longer life time. With the continuous monitoring of the fuel consumption of modern trucks it seems possible to incorporate plasma actuators into an active control loop where they can be activated when needed and that their effect can almost immediately be evaluated.
